# Developing a multi-center clinical data mart of ACEI and ARB for real-world evidence (RWE)

**DOI:** 10.1186/s40885-018-0103-7

**Published:** 2018-12-15

**Authors:** Hun-Sung Kim, Sue Hyun Lee, Tong Min Kim, Ju Han Kim

**Affiliations:** 1 Department of Medical Informatics, College of Medicine, The Catholic University of Korea, 222, Banpo-daero, Seocho-gu, Seoul, 06591 Republic of Korea; 2 Department of Endocrinology and Metabolism, College of Medicine, The Catholic University of Korea, Seoul, Republic of Korea; 3 Division of Biomedical Informatics, Systems Biomedical Informatics Research Centre, Seoul National University College of Medicine, 103 Daehak-ro, Jongno-gu, Seoul, 110-799 Republic of Korea

**Keywords:** Angiotensin-converting enzyme inhibitors (ACEI), Angiotensin II receptor blocker (ARB), Clinical data mart CDM), Real world data (RWD), Real world evidence (RWE)

## Abstract

**Background:**

Randomized controlled trials can be expensive and time-consuming, leading to medical researchers utilizing real-world evidence (RWE) based on already-collected data. We aimed to conduct various RWE studies on angiotensin-converting enzyme inhibitors (ACEI) and angiotensin II receptor blocker (ARB), commonly used as first-line therapy for blood pressure, and to develop a multi-center clinical data mart (CDM) of ACEI/ARB for various clinical purposes.

**Methods:**

Data from electronic medical records of St. Mary’s Hospital and the Seoul National University Hospital were collected. We obtained blood and urine test results of patients within the 30 days prior to their first prescription of ACEI or ARB, as well as the first date of diagnosis and presence of various chronic and cardiovascular diseases using the International Classification of Diseases-10 classification. One researcher managed data quality and collation for each hospital in order to facilitate patient anonymity. When results were unclear, the responsible investigator for each hospital attempted to resolve ambiguities by direct chart review.

**Results:**

A total of 102,333 patients who were prescribed ACEI or ARB for the first time were included (21,481 ACEI, 80,551 ARB, and 301 both). Our ACEI/ARB-CDM included short-term studies (within 12 months) to observe changes in various blood or urinary laboratory test values after the initial prescription of ACEI or ARB and long-term studies to confirm the incidence of various diseases.

**Conclusion:**

We established a CDM of RWE for ACEI/ARB prescription, which included various clinical studies. As we accumulate experience in this process, we expect that the use of RWE research will grow and develop.

## Background

“Big data” as an analysis tool has become a recognized part of medical research. In 2011, McKinsey Global Institute [1] defined big data as datasets whose size is beyond the ability of typical database software tools to capture, store, manage, and analyze. The phrase “big data,” used primarily in the information and communications technologies (ICT) field, has been modified to the phrase “real world data” (RWD) for use in the medical community. RWD is not limited to data from traditional clinical research, and includes data generated from patients’ lifestyles as well as medical information [2, 3]. The U.S. Food and Drug Administration (FDA) [4] defines RWD as data relating to patient health status and/or the delivery of health care routinely collected from a variety of sources and clinical research, for use as real-world evidence (RWE).

Medical researchers are interested in RWE using data already accumulated since randomized controlled trials (RCT) are expensive and time-consuming [5]. The four major medical RWD types include electronic medical record (EMR) data used in hospitals, genomic data, public information data, and lifelog data. Among the four RWDs, the most refined and structured EMR data is recognized as the most reliable data and is comparatively easily accessible. There are disadvantages in that it can be difficult to analyze unstructured data such as freehand text-based notes, but clearly structured data such as imaging data, video, and quantitative laboratory data can also be used [6].

Many medical researchers are attempting RWE studies using EMR data [7–10]. However, if the research hypotheses and design are not clearly defined prior to the beginning of the study, RWE is prone to serious errors or bias, and the results are often unreliable. Data quantity cannot override the importance of ensuring adequate and unbiased control and analysis, and poorly built datasets can be configured based on intrinsically biased designs. We aimed to gather various RWE on the use of angiotensin-converting enzyme inhibitors (ACEI) and angiotensin II receptor blockers (ARB), commonly used as first-line therapy for hypertension [11–13], by building a multi-center clinical data mart (CDM) on ACEI/ARB. The process used to develop this CDM is intended to assist researchers who wish to develop similar clinical data marts for RWE purposes.

## Methods

### Privacy protection and IRB

St. Mary’s Hospital and the Seoul National University Hospital participated in this retrospective cohort study. Data stored in EMRs were used. Because the patients had already been treated, the study did not directly affect the patients. Data on personal information were stored in an encrypted database at each hospital and were accessible only to a designated responsible investigator. Owing to the anonymity of the data and the retrospective nature of the study, informed consent was not required. Patient information was stored after deleting the identifying registration numbers and replacing them with a temporary identifier; the files matching the temporary registration numbers were also encrypted and stored separately. These temporary numbers were accessible only to the responsible investigator at each hospital, who was blinded to patient information outside the designated hospital jurisdiction. The temporary patient numbers were only used when the data were required for integration during statistical analyses. If individual chart reviews of the patients were required, they were requested through the temporary number from the responsible investigator at each hospital, and the registration numbers of the patients were deleted prior to providing the data. This study was approved by the institutional review boards (IRB) of the Catholic University of Korea and the Seoul National University Hospital.

### Definition of EMR extraction by ACEI or ARB

ACEI medication prescribed in the Seoul St. Mary’s Hospital and the Seoul National University Hospital consisted of eight types: Captopril (12.5 mg, 25 mg, or 50 mg), Enalapril (5 mg or 10 mg), Ramipril (2.5 mg, 5 mg, or 10 mg), Lisinopril (10 mg), Imidapril (5 mg or 10 mg), Moexipril (7.5 mg or 15 mg), Perindopril (4 mg or 8 mg), and Zofenopril (7.5 mg, 15 mg, or 30 mg). ARB was prescribed as seven types, including Candesartan (8 mg, 16 mg, or 32 mg), Valsartan (80 mg or 160 mg), Fimasartan (30 mg, 60 mg, or 120 mg), Irbesartan (150 mg or 300 mg), Olmesartan (10 mg, 20 mg, or 40 mg), Telmisartan (40 mg or 80 mg), and Eprosartan (600 mg).

Patient data records were selected on the basis of receiving their first prescriptions of ACEI or ARB. Based on these records, we extracted information including date of birth, height, weight, and systolic and diastolic blood pressure at the time of first prescription. We also extracted hospital information such as the department that first prescribed ACEI or ARB, the last prescribing department, the number of prescription days, and hospitalization status. Blood test and urine test results for blood urea nitrogen (BUN), creatinine, sodium, potassium, uric acid, and other values from within 30 days prior to ACEI or ARB prescription were recorded. If there were more than two test results within the above period, we extracted ACEI or ARB to a value close to the date on which it was first prescribed. We further extracted the first date of diagnosis as well as presence of various chronic and/or cardiovascular diseases [14]. We were able to determine whether the onset of each patient’s disease occurred before or after the first prescription of ACEI or ARB and calculate how many days later the disease recurred. If a diagnosis date was earlier based on the date that the ACEI/ARB was first prescribed, it was included as already diagnosed baseline characteristics. If ACEI or ARB was diagnosed after the date of first prescription, it was defined as the incidence of each disease following the use of ACEI/ARB. The diagnostic name of the extracted disease was assigned using the International Classification of Diseases (ICD)-10 classification, which included hypertensive diseases (I10–15), ischemic heart diseases (I20–25), cerebrovascular diseases (I60–69), aneurysm dissection (I71–73), diabetes mellitus (E10–15), cancer (C-), osteoporosis (M80–82), heart failure (I50, I11.0, I13.0, I24.8), and acute renal failure (N179).

Information on specific prescriptions in addition to ACEI/ARB was also extracted, including date of initial prescription and date of last prescription, and the drug was configured to check whether or not a drug was administered during each study period. Types of medications included β-blockers, calcium channel blockers, diuretics, non-steroidal anti-inflammatory drugs (NSAIDs), and HMG-CoA reductase inhibitors.

### Design

**Fig. 1:**
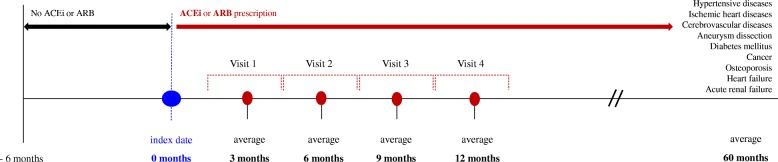
Design of ACEI/ARB-clinical data mart. ACEI, angiotensin-converting enzyme inhibitors; ARB, angiotensin II receptor blocker

Our ACEI/ARB-CDM was designed as short-term studies of 12-month duration to observe changes in various test values such as microalbuminuria or hyperuricemia after the initial prescription of ACEI or ARB, as well as long-term studies to confirm the incidence of various disease (Fig. 1). In contrast with RCT, RWE makes it difficult to accurately predict hospital visits, and actual visit dates are very diverse. For this reason, in this study, the date when a patient was first prescribed ACE was defined as “visit 0.” A subsequent visit 45–136 days later (with a mean of 90 days) was defined as “visit 1”. A subsequent visit 137–227 days later (mean 180 days) was defined as “visit 2,” a visit 228–318 days later (mean 280 days) was defined as “visit 3,” and a visit 319–410 days later (mean 365 days) was defined as “visit 4.” For each visit, information regarding the above measures, prescription changes in ACEI or ARB, and blood and urine test items were collected.

### Data quality management

Data quality management (DQM) is essential for data extracted for EMR-based clinical research prior to statistical analysis. DQM must be performed continuously in order for the extracted data to be used suitable for purpose. In our study, quality control of data values and data structure were simultaneously managed. DQM was conducted by one researcher after integrating data from each hospital. Unstructured laboratory data including characters such as “> 3” and “3+” were deleted, and cases where data management software could not recognize input due to punctuation error were corrected manually. Quality control methods were stored in a standard format for integrating data from the two hospitals into a single dataset. Records of modified data were recorded and the original files were saved separately.

### Direct chart review

In case of DQM of specific data values, such as in unstructured laboratory data containing characters or test results that were not unclear with the patient’s registration number, the responsible investigator at each hospital attempted to directly modify records by reviewing patient charts.

## Results

**Fig. 2:**
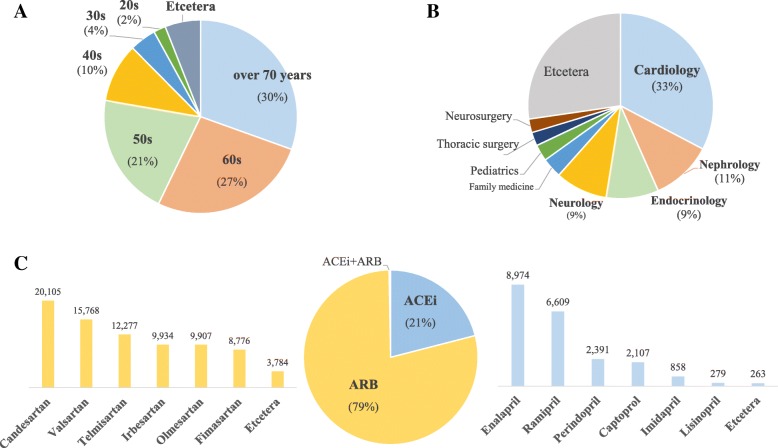
Analysis of ACEI/ARB-clinical data mart (*n* = 102,333) (**a**) age, (**b**) department, (**c**) type of ACEI or ARB. ACEI, angiotensin-converting enzyme inhibitors; ARB, angiotensin II receptor blocker

Between January 1, 2009 and December 31, 2017, a total of 102,333 patients were first prescribed ACEI or ARB at the Seoul St. Mary’s Hospital and the Seoul National University Hospital. This dataset included 56,262 men (55.0%) and 46,069 women (45.0%), as well as two foreign nationals whose gender could not be identified based on chart review. The mean age was 59 ± 28 years, with 31,197 patients (30.5%) being over 70 years old, 27,301 patients (26.7%) in their 60s, 21,038 patients (20.6%) in their 50s, and 10,079 patients (9.8%) in their 40s (Fig. 2a). In 33,455 cases (32.7%), ACEI/ARB was reported by a cardiology department, in 10,921 cases (10.7%) by a nephrology department, in 9380 cases (9.2%) by an endocrinology department, and in 9275 cases (9.1%) by a neurology department. (Fig. 2b).

ACEI was first prescribed in 21,481 cases (21.0%) and ARB was first prescribed in 80,551 cases (78.7%). In 301 cases (0.3%), ACEI and ARB were prescribed at the same time (Fig. 2c). Of the 21,481 patients prescribed ACEI, Enalapril was prescribed to 41.8% (8974/21,481 patients), Ramipril was prescribed to 30.8% (6609/21,481 patients), Perindopril was prescribed to 11.1% (2391/21,481 patients), Captopril was prescribed to 9.8% (2107/21,481 patients), Imidapril was prescribed to 4.0% (858/21,481 patients), and Lisinopril was prescribed to 1.3% (279/21,481 patients). For the 80,551 patients prescribed ARB, 25.0% (20,105/80,551 patients) received Candesartan, 19.6% (15,768/80,551 patients) received Valsartan, 15.2% (12,272/80,551 patients) received Telmisartan, 11.6% (9334/80,551 patients) received Irbesartan, 12.3% (9907/80,551 patients) received Olmesartan, and 10.9% (8776/80,551 patients) received Fimasartan.

A total of 1.3% (1297 /102,333 patients) had been diagnosed with heart failure prior to first prescription of ACEI or ARB and 0.9% (889/102,333 patients) were diagnosed after first prescription. Acute myocardial infarction had previously occurred in 2.2% (2252/102,333 patients) and occurred in 1.0% (1050/102,333 patients) subsequently. Angina pectoris had previously occurred in 8.8% (9050/102,333 patients) and 4.2% (4266/102,333 patients) were diagnosed subsequently. In the case of ischemic heart disease, including angina and acute myocardial infarction, 12.1% (12,409/102,333 patients) were diagnosed before first ACEI/ARB prescription and 5.1% (5179/102,333 patients) were diagnosed subsequently. Cerebrovascular disease, including stroke, was diagnosed in 10.0% (10,191/102,333 patients) before the first prescription and in 3.8% (3877/102,333 patients) after the first prescription. Aneurysm dissection already had previously occurred in 0.9% (931/102,333 patients) and 0.7% (672/102,333 patients) were diagnosed subsequently. Diabetes mellitus was present in 3.9% (3975/102,333 patients) at first prescription and 2.3% (2279/102,333 patients) subsequently. Gout was present in 0.2% (215/102,333 patients) and later diagnosed in 0.4% (431/102,333 patients). Erectile dysfunction had previously occurred in 0.02% (26/102,333 patients) and occurred later in 0.1% (98 /102,333 patients). Cancer had been diagnosed in 8.3% (8463/102,333 patients) and 3.6% (3666/102,333 patients) were diagnosed subsequently. Renal failure had occurred in 0.1% (127/102,333 patients) and 0.2% (180 /102,333 patients) were diagnosed later. Sudden cardiac death occurred in 0.2% (180 /102,333 patients) after their first prescription of ACEI/ARB.

There were 25,753 patients (25.2%) who were prescribed β-blockers, 35,927 (35.1%) prescribed statin drugs, 36,161 (35.3%) prescribed CCB, 30,339 (29.6%) prescribed diuretics, and 6461 (6.3%) prescribed K-sparing diuretics.

## Discussion

The establishment of CDM enables numerous clinical studies with a single construction [15]. We have established a CDM for ACEI/ARB, which includes patient basic data, test data, and diagnosis name. A variety of clinical research is in progress to utilize it.

In cases where a CDM needs to be built for research purposes, baseline characteristics such as systolic blood pressure, diastolic blood pressure, height, and weight that medical staff enter into the EMR manually are extremely important. Data regarding baseline characteristics obtained for this study were not satisfactory, due to numerous missing or incorrectly entered data. In building CDMs, clinical researchers working with data may feel intuitively that a value was entered incorrectly, but it is not permissible to arbitrarily modify it. In some of these instances, a guide or protocol is required to identify incidents where height or weight are reversed or incorrectly entered; in such cases, when the protocol itself is not clearly presented, it can act as an error from the start of the study if this process is shaken. Ideally, data directly entered by medical staff should only be corrected by the staff member in question during the chart review process. However, the biggest strength of the EMR-based research over RCT is that data can be extracted in a short time, and this advantage can be lost if frequent chart reviews are made. Moreover, it is virtually impossible to review chart data piece by piece if there are more than a few thousand large CDM deployed. All told, for effective CDM building, large numbers of patients must be included, making human error nearly inevitable and predisposing studies to bias. This emphasizes the importance of advance anticipation of such issues and the establishment of written protocols and guidance prior to the commencement of clinical research.

The most clear and reliable data points in EMR databases come from blood testing [16], with such structured objective data tending to have fewer errors. Moreover, since the normal range of test results is clearly defined, most RWE studies rely on laboratory data [16–18]. This is the most reliable data for estimating incidence of adverse drug reactions [19–21]. However, it is necessary to define missing data that are outside of the measurable range of equipment. These may be reported as “< 0.3” or “higher than 18,” or may be entered as missing data. As before, it is important to establish clear protocols for dealing with these instances prior to research and a significant amount of data loss may be inevitable [22]. This may also require manual work through direct chart review.

The next most clear and structured form of data are the specific diagnoses for diseases. ICD-9 or ICD-10 classification is suitable for this purpose. For example, it is possible to extrapolate the correlation between a specific drug and a specific diagnosis in order to determine whether a specific diagnosis occurred before or after the drug is administered. In addition, it is possible to calculate the interval from the first administration of a specific drug to the first date that a specific disease was diagnosed. For example, it is straightforward to conduct studies into the difference in the incidence of diabetes according to the prescription of statin, a dyslipidemia drug [23]. Here again, the biggest hurdle is omitted diagnosis by the medical staff. There may be a difference between a patient’s actual diagnosis and the diagnostic name (billing or claiming data) that medical staff enter manually. In practice, medical staff are often passive in adding new diagnostic names [24]. Severe concerns such as cancer and myocardial infarction tend to have a clearer diagnosis, but milder concerns such as common cold, nausea, or constipation are more likely to be omitted and unreliable. As such, researchers should be aware of the potential for underestimation when conducting studies of specific diagnostic nomenclature.

RWE studies are the most advantageous to evaluate side effects with low incidences [25], as side effects with lower incidence are less likely to be observed in RCTs but can be easily identified within a large CDM of patients who have already been treated. Using our CDM, we conducted a study on ARB-related angioedema with a low incidence of expression [26], which indicated that it is significantly overlooked by medical staff. In fact, if it is a side effect from the experience of the medical staff, not from the side effects of the examination or clear structured laboratory data, follow-up research may be difficult due to incomplete chart records. This is why many studies have been conducted on data standards recently.

The data mart built in this study includes data on all patients who were first prescribed ACEI/ARB. There was no manipulation of data, except for that described in the Methods section. The Reviewer is well aware of the manipulation and the fact that it was inevitable due to missing data for each item (because of the differences in the tendency of the physician to record data and the characteristics of each division). For example, in this study, the missing data for BMI were as high as 55.7% (56,981/102,333), because BMI data had to be entered manually. In contrast, missing data on laboratory blood tests were low because of the structured nature of the data. Nevertheless, for laboratory blood test data, the distribution of missing data varied for each test item, according to the characteristics of each division or physician. If researchers want to conduct studies related to ACEI/ARB, they can use this data mart, and depending on the purpose of the study, each item that they want to extract within the same data mart will be different. The researcher should be clearly aware of this before proceeding with the study. In other words, the data required from the data mart will vary depending on the purpose of the study. Therefore, even if there are missing data while building a data mart, manipulation or supplementation by selection, exclusion, addition, and deformation should not be performed. Researchers should be aware of the missing data already and choose appropriately to meet the purpose and hypothesis of their studies.

Our team has conducted several studies on RWE and has built various CDMs [8, 15, 19, 26], but many methodological errors remain to be corrected. In many existing RWEs, it is often the case that a large sample size offers an appealing advantage over RCTs. However, unlike RCTs, which aim for group homogeneity, RWDs do not provide a lot of information due the heterogeneity of the included patient data [27]. An important aspect to be addressed in the future is the need for standardized protocols, mainly in terms of data extraction: data should exist in a well-structured form and should be easy to extract. However, the current EMR system unfortunately does not meet these needs. Therefore, in order to extract the correct data without missing data in the future, standardized protocols for input and preparation, standardized by researchers, clinics, and hospitals, will be needed. In other words, to eliminate missing data, the data should be collected in a structured format for easy entry. Second, from the standpoint of medical staff utilizing extracted data, standardized protocols for data utilization are required. This is because, depending on how researchers utilize their data (or depending on the statistical approach), it may lead to varying results. These two aspects mentioned above are also the limitations of RWE, which have to be overcome and will not be easy, because EMR data are not intended for clinical research and for use to develop data marts. Several concerns remain regarding data collection to develop data marts, as this area of research is still in the developmental stage. Nevertheless, it is natural that we should strive to minimize missing data, and it is possible to minimize biases only by conducting research using stringent and well-designed protocols.

## Conclusions

RWE is already recognized as a novel clinical research method, and its influence is enormous. However, it does not challenge to the strengths of RCT, and instead may be valued as a study preceding RCT. RCT and RWE are complementary and we expect that their use in conjunction can compensate for the disadvantages of conventional RCT. Nonetheless, even using large sample sizes, there are many questions that cannot be answered with confidence and will need to be continually attempted and supplemented. In this study, we have established a CDM for RWE regarding ACEI/ARB therapy and expect various clinical studies to be available accordingly. As we accumulate experience in this process, we expect that much of RWE research will grow and develop.

## Abbreviations

ACEI: Angiotensin-converting enzyme inhibitors
ARB: angiotensin II receptor blocker
CDM: clinical data mart
DQM: Data quality management
EMR: Electronic medical record
GFR: Glomerular filtration rate
ICD: International Classification of Diseases
IRB: Institutional review boards
MDRD: Modification of diet in renal disease
RCT: Randomized controlled trial
RWD: Real world data
